# JD419, a *Staphylococcus aureus* Phage With a Unique Morphology and Broad Host Range

**DOI:** 10.3389/fmicb.2021.602902

**Published:** 2021-04-22

**Authors:** Tingting Feng, Sebastian Leptihn, Ke Dong, Belinda Loh, Yan Zhang, Melanie I. Stefan, Mingyue Li, Xiaokui Guo, Zelin Cui

**Affiliations:** ^1^Department of Clinical Pharmacy, Shanghai General Hospital, Shanghai Jiao Tong University School of Medicine, Shanghai, China; ^2^Zhejiang University-University of Edinburgh Institute (ZJU-UoE), Zhejiang University, Haining, China; ^3^Key Laboratory of Parasite and Vector Biology, Ministry of Health, Shanghai, China; ^4^School of Global Health, Chinese Center for Tropical Diseases Research, Shanghai Jiao Tong University School of Medicine, Shanghai, China; ^5^Centre for Discovery Brain Sciences, The University of Edinburgh, Edinburgh, United Kingdom; ^6^Department of Pathology and Laboratory Medicine, Perelman School of Medicine, University of Pennsylvania, Philadelphia, PA, United States; ^7^Department of Laboratory Medicine, Shanghai General Hospital, Shanghai Jiao Tong University School of Medicine, Shanghai, China

**Keywords:** *Staphylococcus aureus*, bacteriophage, morphology, genome, host-range, phage therapy

## Abstract

Phage therapy represents a possible treatment option to cure infections caused by multidrug-resistant bacteria, including methicillin and vancomycin-resistant *Staphylococcus aureus*, to which most antibiotics have become ineffective. In the present study, we report the isolation and complete characterization of a novel phage named JD219 exhibiting a broad host range able to infect 61 of 138 clinical strains of *S. aureus* tested, which included MRSA strains as well. The phage JD419 exhibits a unique morphology with an elongated capsid and a flexible tail. To evaluate the potential of JD419 to be used as a therapeutic phage, we tested the ability of the phage particles to remain infectious after treatment exceeding physiological pH or temperature. The activity was retained at pH values of 6.0–8.0 and below 50°C. As phages can contain virulence genes, JD419’s complete genome was sequenced. The 45509 bp genome is predicted to contain 65 ORFs, none of which show homology to any known virulence or antibiotic resistance genes. Genome analysis indicates that JD419 is a temperate phage, despite observing rapid replication and lysis of host strains. Following the recent advances in synthetic biology, JD419 can be modified by gene engineering to remove prophage-related genes, preventing potential lysogeny, in order to be deployed as a therapeutic phage.

## Background

Antimicrobial resistance (AMR) presents a significant threat to humankind. A study estimates that in 2050 about 10 million people will die annually from untreatable bacterial infections if alternatives and novel antibiotics are not available (O’Neill, 2014). The rate of antibiotic-resistance in bacteria has been increasing over the decades due to the over-and misuse of antibiotics (Leptihn, 2019). So-called antimicrobial stewardship programs aim to optimize antibiotic use to decrease the emergence and spread of infections caused by multidrug-resistant organisms (Manohar et al., 2020b). Due to this and the lack of financial incentives for the pharmaceutical industry to develop new antibiotics, alternative treatment strategies are urgently needed. One such strategy is the deployment of bacterial viruses that infect and kill bacteria, also known as phage therapy (Manohar et al., 2020a). Phage therapy has been used successfully against many Gram-negative and -positive bacteria, including *S. aureus* but is not yet a standard therapeutic strategy (Abedon et al., 2011; Schooley et al., 2017; Maddocks et al., 2019). *S. aureus* is a particularly concerning pathogen as it exists as a harmless commensal. Yet, it can cause various types of infections, from skin abscesses to lethal bloodstream infections, often in hospitals. Phage therapy shows promising potential for treating methicillin- as well as vancomycin-resistant *S. aureus* (Moellering, 2012; Limbago et al., 2014) as phages can infect multidrug-resistant bacteria (Lobocka et al., 2012; Kazmierczak et al., 2014).

While members from other families have been reported, most *Staphylococcus* phages such as K, Twort, CH15, φSA012, etc. belong to the family of *Myoviridae*, which in some instances exhibit a broad-host-range that is considered beneficial for phage therapy (Cui et al., 2017b; Iwano et al., 2018). *Podoviridae* infecting *S. aureus* have also been reported (Culbertson et al., 2019) and have been used together with other phages in so-called phage cocktails (“Microgen cocktail”) for the treatment of wound and skin infections (McCallin et al., 2018; Kornienko et al., 2020). *Staphylococcus* phages belonging to the family of *Siphoviridae* have been found worldwide; however, many of them display a lysogenic life cycle while occasionally also carrying virulence genes and antibiotic-resistant genes, which are the undesired characteristics for therapeutic phages. Several reports have shown the potential use of *Staphylococcus* phages belonging to *Siphoviridae* alone or combined with chemical antibiotics to penetrate and remove biofilms to treat infections (Rahman et al., 2011; Ji et al., 2020).

With recent advancements in genome engineering, lysogenic phages represent a valuable therapeutic virus source and have been recently employed in clinical therapy (Dedrick et al., 2019). While many lytic phages have been characterized in the past, considerably fewer lysogenic phages have been described on the biochemical level due to their lifestyle complexity, with the exception of prophage sequence analyses in bacterial genomes (Goerke et al., 2009; Loh et al., 2020a) or the impact of prophages on their hosts (Ingmer et al., 2019). When properly characterized, lysogenic phages can be a treasure-trove for genetically modified therapeutic phages. In this work, we describe *Staphylococcus* phage JD419, a comparably “unstable lysogenic phage” as lysogeny seems not to be preferred, as the phage quickly undergoes replication and host cell lysis. The morphology of JD419 revealed that the phage belongs to the *Siphoviridae*. Whole-genome analysis of the *Staphylococcus* phage indicates that it does not contain any virulence factors nor antimicrobial resistance genes, which must be absent in therapeutic phages. Due to a remarkable killing ability and broad host range of JD419, this phage is an ideal target for genetic engineering to create a lytic phage by removing genes allowing integration and gene repression following an approach similar to Dedrick et al. (2019), as our study illustrates that *Staphylococcus* phage JD419 has the potential to be a high-value therapeutic phage for prophylaxis and therapeutic purposes.

## Materials and Methods

### Bacteria Isolates and Culture Conditions

A total of 135*S. aureus* isolates were obtained from the clinical samples, including blood, wound secretions, and sputum from patients with *S. aureus* infections in different hospitals in Shanghai, China. Strains were identified by the VITEK 2 Compact (Biomerieux); 76 strains were MSSA (methicillin-sensitive *Staphylococcus aureus*), 59 strains were MRSA (Methicillin-resistant *Staphylococcus aureus*), which were tested for resistance to cefoxitin; detailed information can be found in the Supplementary Material. Three standard strains, including N315, USA300, and Newman, which Han Yang kindly provided in Shanghai General Hospital, were also used. Isolates were grown in liquid LB (Luria-Bertani) medium at 37°C, on LB medium (1.5% agar), or in LB soft agar overlays (0.7% agar) (Sangon Biotech (Shanghai) Co., Ltd, China). The phage JD419 was initially found as plaques in the lawn of *S. aureus* clinical isolates MR-84 belonging to MLST 239 type, which was observed in the Molecular Microbiology laboratory in Shanghai Jiao Tong University School Medicine, Shanghai, China. Even without any chemicals such as Mitomycin C, clear plaques were observed.

The phage was purified three times from plaques using two-layered agar plates described previously (Cui et al., 2017a). High-titer phage stocks (2 × 10^9^pfu/mL) were obtained through amplification in liquid LB medium containing 10 mM MgCl_2_ and 5 mM CaCl_2_ (Sangon Biotech (Shanghai) Co., Ltd.) (Cui et al., 2017a), for which *S. aureus* MR-84 was used as a host strain. 101 of 138 different strains of *S. aureus* were characterized using the MLST typing method, as previously described (Bao et al., 2013).

### *Staphylococcus* Phage JD419 Amplification and Purification

First, phage JD419 was added to host cells of *S. aureus* strain MR-84 at a Multiplicity of Infection (MOI) of 0.01. After incubation at 37°C overnight, cell lysis -indicated by a decrease in the liquid culture’s turbidity- was observed. The lysate was then incubated with chloroform (final concentration was 2%) for 30 min under gentle shaking to kill the residual bacteria and release phage particles from bacterial cells. Enrichment and purification of phage particles were conducted as described previously (Cui et al., 2017a). Briefly, cell debris was removed by centrifugation at 6,500 rpm (Beckman, JA18.0) for 15 min, followed by the precipitation of phages in the supernatant using polyethylene glycol (PEG) 8000 (at 4°C overnight) and precipitated (final concentration 10% w/v) by centrifugation at 8,500 rpm, for 20 min (Beckman, JA18.0). The pellets were re-suspended in TM buffer [(Tris-Mg^2+^ Buffer) 10 mM Tris–HCl (pH 7.5), 100 mM NaCl, 10 mM MgCl_2_, 5 mM CaCl_2_)], vortexed, and PEG8000 was removed by adding the same volume of chloroform and vortexed, centrifuged at 4,000 *g*/min for 10 min. The supernatant contained high concentrations of phage to which CsCl was added (0.5 g/mL). The phages were then purified by centrifugation in a CsCl gradient (1.33, 1.45, 1.50, 1.70 g/cm^3^) in TM buffer in Ultra-Clear tubes (Beckman Coulter Inc., Fullerton, CA, United States). Finally, the band containing the enriched phages was taken out using a syringe, then dialyzed against TM buffer and stored at 4°C.

### Electron Microscopy Imaging

After centrifugation in a CsCl gradient (described above), purified phage particles obtained by dialysis were collected by centrifugation at 33,000 × *g* for one hour (Beckman high-speed centrifuge and a JA-18.1 fixed-angle rotor) and washed twice in 0.1 × PBS (pH 7.4). The sample was deposited on carbon-coated copper grids and stained with 2% (w/v) potassium phosphotungstate (pH7.0). Phage photos were taken using a Hitachi H7500 transmission electron microscope (TEM) at 80 kV.

### Host Range Determination

The host range of phage JD419 was analyzed by spotting serial dilutions of the phage on a two-layer soft agar lawn of *S. aureus*. Two microliters of a dilution series of phage lysate (≈10^8^ PFU/ml) were gently pipetted on a Luria-Bertani (LB) medium plate overlay agar plate containing *S. aureus* (overlaid with *S. aureu*s (OD_600_≈0.4) mixed in 0.7% top agar and cultured 30 min). Phage’s lytic ability was assessed based on the clarity of the inhibition zone as published previously (Cui et al., 2017a): clear, turbid, or faint. Lytic activity was confirmed by determining the phage’s ability to create single plaques after incubating serial dilutions of the phage with host bacteria using the soft-agar overlay technique. 138 strains of *S. aureus* were used to establish an estimate of the host range of phage JD419.

### Determination of Adsorption Rate

Phage JD419 was added to an exponentially growing culture of *S. aureus* MR-84 at an MOI of 0.0005 and incubated at room temperature without shaking. An aliquot of 100 μL, containing a mixture of phage JD419 with *S. aureus* MR-84, was taken at different time points (1–30 min) 1, 3, 5, 7, 9, 12, 15, 18, 21, 24, 27, and 30 min respectively, and centrifuged immediately at 16,000 *g* for 30 s once collected. Afterward, the titers of phage JD419 in the supernatant were determined by counting the plaques on overlay agar plates. The adsorption rate of phage JD419 to the *S. aureus* MR-84 at the time of i was calculated by the formula of (N_0_-N_*i*_)/N_0_^∗^100, with N_*i*_ representing the titer of phage in the supernatant after co-incubation for i minutes, and N_0_ is the titer of phage before co-incubation. The proportion of non-adsorbed phages to the number of phages used for infection and standard deviations were calculated.

### One-Step Growth Curve and the Burst Size of *Staphylococcus* Phage JD419

*Staphylococcus aureus* cells at mid-log growth phase were harvested by centrifugation at 13,000*g* for 15 min and resuspended in fresh LB medium. Phage JD419 was added to the culture at an MOI of 0.01 and allowed to adsorb for 10 min at room temperature, then incubated at 37°C with gentle shaking. Aliquots were taken at different points (1–110 min) 1, 5, 10, 15, 20, 25, 30, 35, 40, 45, 50, 55, 60, 65, 70, 75, 80, 85, 90, 100, and 110 min. After centrifugation, the phage titers were determined by counting plaques on overlay agar plates.

Phage JD419 was added to *S. aureus* MR-84 at an MOI of 0.01. The burst size was calculated using the formula (N_*f*_-N_0_)/N_0_, where N_*f*_ represents the titer of phage JD419 at the beginning of the next growth circle, and N_0_ represents the titer of phage JD419 at the beginning of the former growth circle.

### Stability of *Staphylococcus* Phage JD419

The pH stability of phage JD419 was assessed as follows: 100-fold dilutions of the initial phage solution containing 10^8^ PFU/mL were added to TM buffer with pH values ranging from 2 to 12 incubated at 37°C for 1 h. Subsequently, the samples were diluted in TM buffer (pH 7.5), and the titer of each sample was determined. To address thermal stability, phage JD419 were incubated in TM buffer (pH7.5) at different temperatures (24°C, 37°C, 40°C, 50°C, 60°C, and 70°C) for 1 h, then the titer was determined.

### Inhibition Growth Curve of *Staphylococcus* Phage JD419

Four milliliters of TSB (Oxiod; containing 5mM CaCl_2_ and 10mM MgSO_4_) in an L-shaped plastic test tube was inoculated (1%) with an overnight *S. aureus* (either N315 or MR-84) culture, *Staphylococcus* phage JD419 was added at an MOI of 100, 10, or 0 and incubated at 37°C without shaking. OD600 value at different time points (interval 30 min extending 8 h) was measured. Furthermore, an independent experiment involved the CFU of *S. aureus* in the group of MOI = 100 and 0 being counted by serial dilution of the mixture using the CFU forming assay in the TSA plate (TSA, Oxiod); they were measured by interval 2 h extending 12 h.

### Genome Analysis

The DNA of *Staphylococcus* phage JD419 was extracted using an Aidlab kit (Aidlab Biotechnologies Co., Ltd, Beijing, China). The complete genome of *Staphylococcus* phage JD419 was obtained by 454 pyro-sequencing at the Chinese National Human Genome Center (Shanghai, China). Assembly of quality filtered reads was performed using the 454 Life Sciences Corporation platform. The sequence of JD419 was deposited under the nucleotide sequence accession number MT899504 (GenBank). tRNAs were detected using tRNAscan-SE software (Lowe and Eddy, 1997). To identify any virulence factors or antibiotic-resistant genes in the genome of *Staphylococcus* phage JD419, Blast searches were performed against the antibiotic resistance genes database (ARDB)^1^ (Liu and Pop, 2009) and the virulence factors database^2^. Genes with more than 70% coverage and a minimum identity of 30% were retained as results. The software PHACTS was used to determine whether *Staphylococcus* phage JD419 is a lytic or lysogenic (temperate) phage (McNair et al., 2012). Comparisons of complete genome sequences of other phages were performed with Mauve20150226 (Darling et al., 2010); Neighbor-joining trees were constructed using the major capsid proteins to analyze phylogeny JD419 by MEGA5. ORFs were predicted using several algorithms, including those of the software glimmer, RAST, and GeneMarkS (Delcher et al., 1999; Besemer and Borodovsky, 2005; Aziz et al., 2008). A two-tailed unpaired Student’s t-test was used for statistical analysis with GraphPad Prism Software and R. *p*-values of less than 0.05 were considered significant unless specifically indicated otherwise.

## Results

### Phage JD419 Has a Flexible Tail Attached to a Prolate Capsid

**FIGURE 1:**
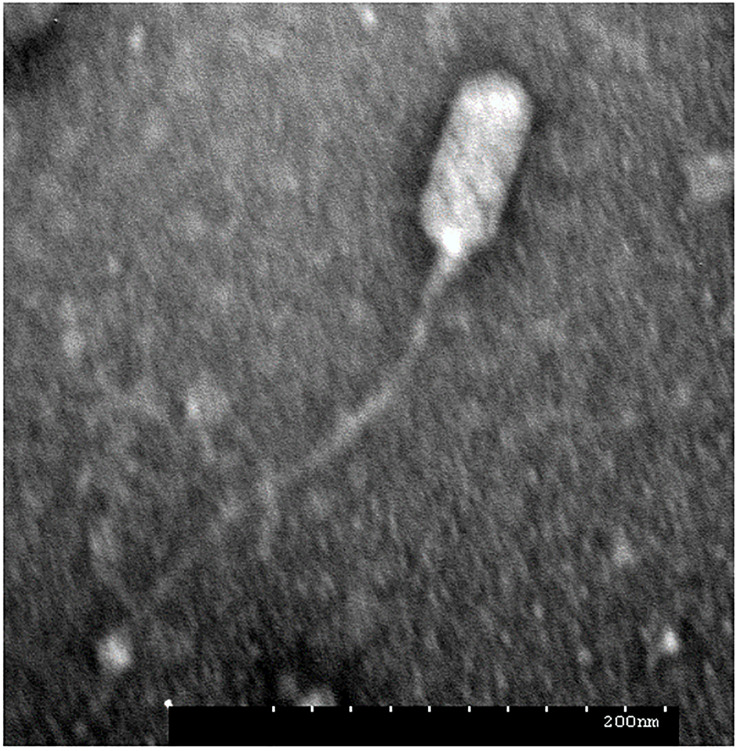
Morphology of *Staphylococcus* phage JD419. The electron micrograph of phage JD419 shows that the tail is flexible and has a length of about 300 nm. The head of the phage is prolate, i.e., extended. The minor axis semi-diameter and major axis semi-diameter of its head are about 50 and 100 nm. Bar: 200 nm.

During routine culture of clinical *S. aureus* strains (Supplementary Table 1), plaques were observed in the lawn of isolate MR-84. Phages often occur together with their host, either as prophages or in natural prey-predator situations. The phage termed JD419 was isolated from the plaques and further purified three times. To address the morphology phage JD419 displays, we employed Transmission Electron Microscopy (TEM). Electron micrographs show that the tail is about 300 nm in length, while the head has a prolate geometry, elongated only in one direction. The length to width ratio is approximately 2, with a length of 100 ± 5 nm and a width of 50 ± 2 nm (Figure 1). A tail sheath between the head and tip of the tail is absent. The overall morphology indicates that phage JD419 is a member of the family *Siphoviridae* in the order Caudovirales, however, with an unusually elongated head. Table 1 lists phages with similar morphology described in the literature, which infect different bacteria or exhibit distant genetic relationships to JD419.

**TABLE 1:** Sightings of phages with similar morphology to *Staphylococcus* phage JD419.

Phage	Host	Head diameter (nm)	Tail length (nm)	References and comments
IME-EF1	*E. faecalis*			Zhang et al., 2013
P70	*L. monocytogenes*			Schmuki et al., 2012
3A	*S. aureus*	50 × 100	350	http://www.phage.ulaval.ca
JD419	*S. aureus*			This study
4CbK	*C. crescentus*	50 × 200	300	Leonard et al., 1972

### The Adsorption Rate of Phage JD419 to the Host Surface Is a Rapid Process

**FIGURE 2:**
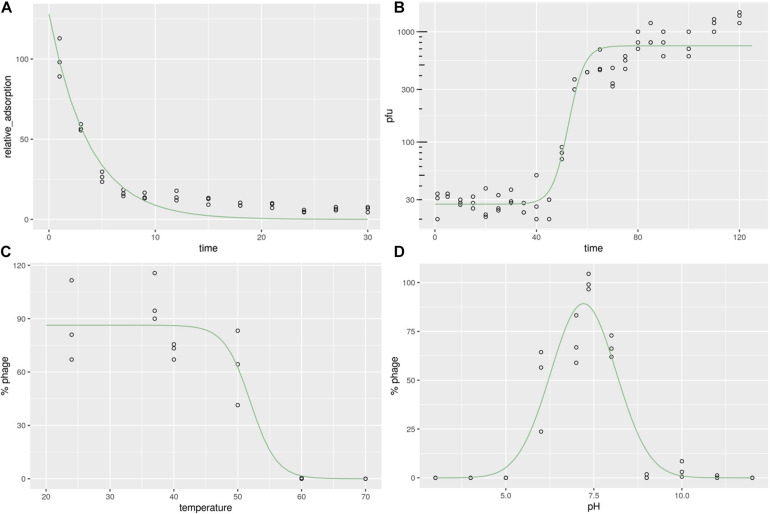
Characterization of *Staphylococcus* phage JD419. **(A)** Adsorption rate of *Staphylococcus* phage JD419 to its host *S. aureus* MR-84. The *X*-axis represents the incubation time of *Staphylococcus* phage JD419 and its host, and the *Y*-axis represents the percent of phage that its host had not absorbed **(B)** One-step growth curves of *Staphylococcus* phage JD419; *X*-axis represents co-culture time of *Staphylococcus* phage JD419 and MR-84, *Y*-axis represents the change in titer of phage JD419 in the mixture. **(C)** Thermal stability of phage JD419; *X*-axis represents different temperatures; *Y*-axis represents the relative titer of *Staphylococcus* phage JD419 to that the titer at 37°C;% stability = (N/N_0_) × 100, where N was the number of viable phages after one hour of incubation and N_0_ was the initial number of phages. **(D)** pH stability of *Staphylococcus* phage JD419; *X*-axis represents the different pH values; *Y*-axis shows the percentage stability relative to the PFU/mL at pH 7.45.

To calculate the phage adsorption rate to its bacterial host, we measured the time-dependent adsorption of phages to bacterial cells by simple co-incubation and subsequent counting of phage particles present in the supernatant. After incubation with its host strain MR-84 for 10 min, more than 80% of phages adhered to the bacterial surface, while after 20 min, more than 90% of phages remained attached to the host (Figure 2A). The observed adsorption rates could be fitted to an exponential decay reaction, with half of the phages attaching to the host cell within approximately 3 min, demonstrating the rapidity of this reaction, indicating a high affinity between phage and host.

### Phage Replication of JD419 Requires More Than the Typical Generation Time of the Host, Releasing About 30 Progeny Particles

When JD419 was used to infect a growing culture of the *S. aureus* host strain MR-84 with an MOI of 0.01, we observed phage particles released after a latent period of approximately 50 min. Complete lysis of all cells was observed at around 70 min. This data was fitted using a sigmoidal fit, showing that half of the phages have been fully released from the cells within 50 min, indicating that the host requires more than one generation time commonly observed for its host, *S. aureus*, when not infected by phages (Figure 2B). We also measured the number of phage particles that were released per cell. Here, we could determine a burst size of approximately 33 phages produced on average per cell.

### Stability of Phage JD419 at Elevated Temperatures and Non-neutral pH Values

For a phage to be deployed for therapeutic use, the stability of the phage needs to be considered. Thermal stability assays show that JD419 remains an active infectious particle between 24 and 40°C (*p* > 0.05). By comparison, almost all phages were inactivated when incubated at 60°C for 1 h (*p* < 0.05) (Figure 2C). When data points were fitted with a sigmoidal function, the point at which half of the phages were inactivated lies at approximately 52°C (± 2.36).

The inactivation in basic or acidic solutions was measured within a pH of 3 to 12. Phage JD419 was stable at pH values between 6 and 8 (Figure 2D), with calculated optimum stability at pH 7.2 (± 0.073), using a Gaussian fitting function. Its stability makes JD419 suitable for most therapeutic applications except the oral route, as the stomach’s acidic environment would inactivate the phage.

### Phage JD419 Exhibits a Broad Host Range

**FIGURE 3:**
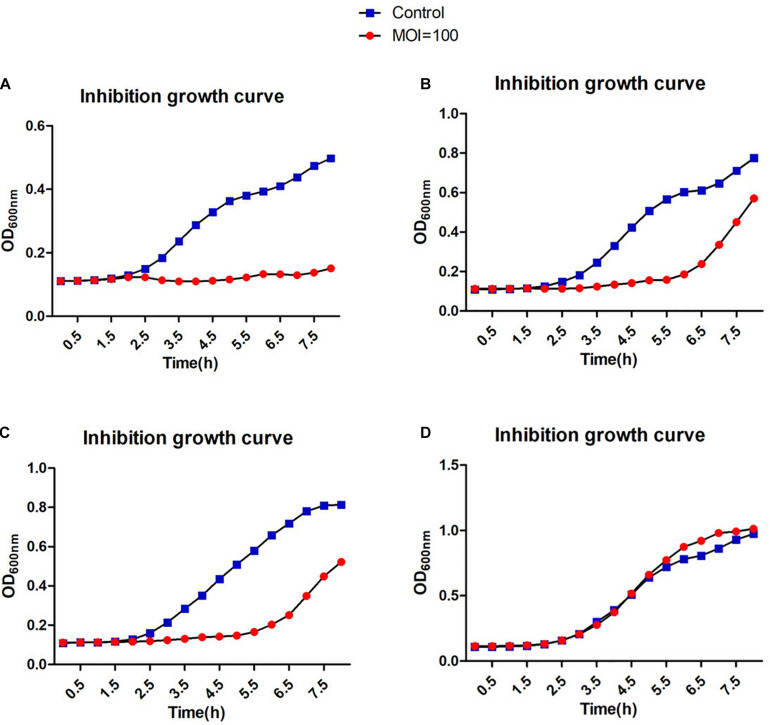
Inhibition assays of *Staphylococcus* phage JD419. The *x*-axis represents the co-culture time of phage JD419 and different strains of *S. aureus*, and the *y*-axis represents the change in *OD*_600_ in the mixture of *Staphylococcus* phage JD419 infecting different strains at the MOI = 100. Panel **(A)** represents strain N315; panel **(B)** represents strain MR-84; panel **(C)** represents strain MS-41; panel **(D)** represents strain MS-57.

**FIGURE 4:**
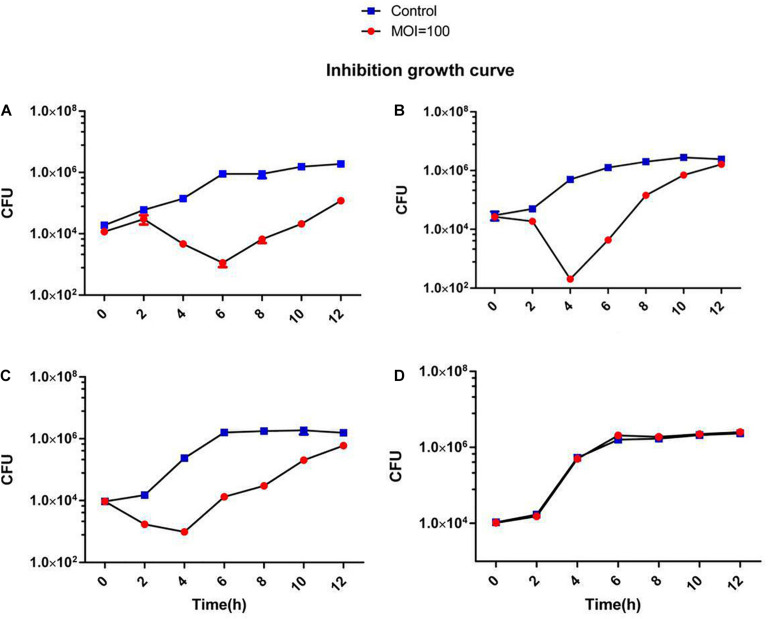
Inhibition assays of *Staphylococcus* phage JD419. The *x*-axis represents the co-culture time of phage JD419 and different strains of *S.aureus*, and the *y*-axis represents the change in CFU (5uL) in the mixture of *Staphylococcus* phage JD419 infecting different strains at the MOI = 100. Panel **(A)** represents strain N315; panel **(B)** represents strain MR-84; panel **(C)** represents strain MS-41; panel **(D)** represents strain MS-57.

A broad host range would allow a therapeutic phage to be used on a large number of different strains. Therefore, it is advantageous to discover phages that can infect many clinical strains. We screened 138 different clinical *S. aureus* strains from two different hospitals in Shanghai, China. Many of the methicillin-resistant strains were classified according to their MLST types and included the prevalent ST239 and ST59 (Supplementary Figure 1). Phage JD419 can lyse 44.2% of tested strains (61/138). However, the extent of lysis was not identical for all strains. We categorized the phage host-range according to the visual appearance of single plaques (Supplementary Figure 1). While some of the strains exhibited plaque-like clearance zones when spotting phage lysate on lawns (albeit having a turbid appearance), some of these did not allow the formation of single plaques in an overlay assay, and these strains were therefore labeled as non-susceptible. The ability to infect and lysis efficiency showed no correlation with the MLST types (Supplementary Table 1). In addition to plaque formation, we also used four isolates of *S. aureus* to investigate the impact on growth in the presence of JD419. Here, we observed that at an MOI of 100 and 10 (Supplementary Figure 1), the growth of three phage-susceptible strains (N315, MR-84, MS-41) was inhibited, while a non-susceptible strain grew similar to the control in which phage was absent (Supplementary Figure 1 and Figures 3, 4).

### Genome Analysis of *Staphylococcus* Phage JD419

**FIGURE 5:**
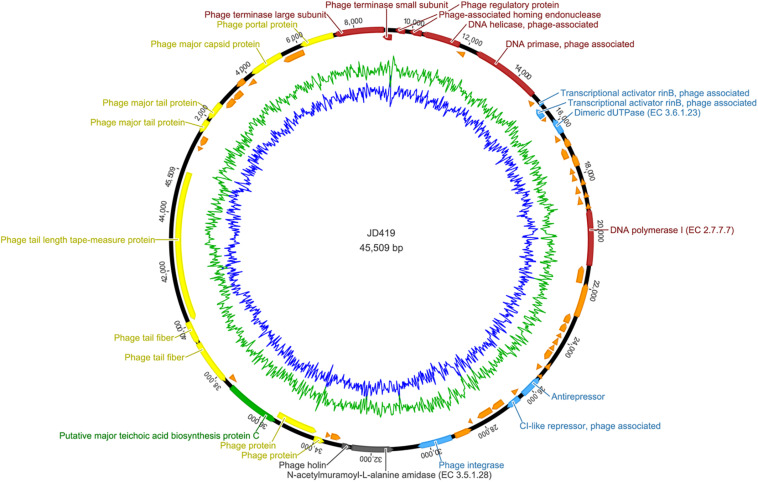
Complete genome sequence of *Staphylococcus* phage JD419. Genome map shows the predicted ORFs; arrows indicate the direction of transcription, proposed functional modules have been marked based on BLAST and domain search results.

To assess the potential of phage JD419 to be used in phage therapy for treating *S. aureus* infections, we sequenced the complete genome of the bacterial virus. Our analysis showed that the phage has a circular genome with 45509 bp. Phage JD419 contains 65 putative open reading frames (ORFs). Most of the proposed ORFs have the same orientation, and several genes overlapped. Putative functions of the ORFs were assigned based on BLASTP analysis and domain searches. We categorized ORF encoded proteins into five modules: structure, DNA synthesis, lysis, regulation, and metabolism (Table 2 and Figure 5).

**TABLE 2:** Predicted functional ORFs of *Staphylococcus* phage JD419.

ORFs	Start	Stop	Strand	Function
JD419_ORF3	1889	1434	−	Phage major tail protein
JD419_ORF4	2622	1981	−	Phage major tail protein
JD419_ORF9	5305	4142	−	Phage major capsid protein
JD419_ORF10	6090	5317	−	Prophage Clp protease-like protein
JD419_ORF11	7312	6074	−	Phage portal protein
JD419_ORF12	9008	7317	−	Phage terminase large subunit
JD419_ORF13	9303	8998	−	Phage terminase small subunit
JD419_ORF14	9748	9428	−	Phage-associated homing endonuclease
JD419_ORF15	10342	9905	−	Phage regulatory protein
JD419_ORF16	11713	10355	−	DNA helicase, phage-associated
JD419_ORF18	14781	12334	−	DNA primase, phage associated
JD419_ORF20	15253	15101	−	Transcriptional activator rinB, phage associated
JD419_ORF21	15636	15250	−	Transcriptional activator rinB, phage associated
JD419_ORF23	16412	15876	−	Dimeric dUTPase
JD419_ORF35	21200	19239	−	DNA polymerase I
JD419_ORF47	26300	25524	−	Antirepressor
JD419_ORF49	26702	27016	+	CI-like repressor, phage associated
JD419_ORF54	29205	30410	+	Phage integrase
JD419_ORF55	32706	31252	−	Phage lysin, N-acetylmuramoyl-L-alanine amidase
JD419_ORF56	33020	32718	−	Phage holin
JD419_ORF61	37423	35513	−	Putative major teichoic acid biosynthesis protein C
JD419_ORF63	39312	37729	−	Phage tail fiber
JD419_ORF64	40145	39321	−	Phage tail fiber
JD419_ORF65	45499	40145	−	Phage tail length tape-measure protein

Genes encoding for structural proteins, such as the major tail protein, the major capsid protein, a portal protein, tail fiber proteins, and a tail length tape-measure protein, were identified. ORFs involved in DNA replication including DNA helicase, DNA primase, DNA polymerase I, phage-associated homing endonuclease, phage terminase large subunit, phage terminase small subunits, phage integrase, a phage-associated homing endonuclease, and a gene with homology to a phage integrase were identified. Genes of holin and lysin (an *N*-acetylmuramoyl-L-alanine amidase) were also identified, both crucial for lysis. We found ORFs that might influence metabolic processes in the host. These genes coded, for example, a dimeric dUTPase, and a putative major teichoic acid biosynthesis protein C. We also identified ORFs involved in regulation, including the transcriptional activator rinB, CI-like repressor, and a prophage Clp protease-like protein, indicating JD419 is a temperate phage. According to the algorithm of the program PHACTS (on January 10, 2021), JD419 phage was confidently predicted as having a temperate lifestyle (Averaged Probability 0.694, Standard Deviation 0.043). While we could confirm that the genome of JD419 does not contain any known virulence factors, we were unable to identify putative roles or functions of a total of 40 of the 65 ORFs encoded proteins, as search algorithms solely reveal that the putative proteins had unknown functions. tRNAs, occasionally found in other large phages such as T4, have not been identified in the genome of JD419.

## Discussion

The clinical use of suitable phages in treating infections might present a potential solution to the threat posed by antibiotic-resistant bacteria. *S. aureus*, which can exist as a harmless commensal bacterium, can cause severe and lethal infections that become increasingly difficult to treat, as many strains have acquired multidrug resistance. Bacteriophages can be used to treat infections caused by such strains, aiming to inactivate the pathogen if antibiotics are ineffective as a treatment. As in most cases, there is no sufficient time to isolate phages that can infect the particular strain that causes the infection; therefore, it is ideal to have phages that exhibit a broad host range at one’s disposal. In this work, we characterized the phage JD419, which can infect clinical strains of *S. aureus*. Its morphology is similar to other reported bacteriophages, such as IME-EF1, P70, and 3A (Leonard et al., 1972; Schmuki et al., 2012; Zhang et al., 2013). However, in general, phages with such elongated capsids and a flexible tail have rarely been described in the literature.

**FIGURE 6:**
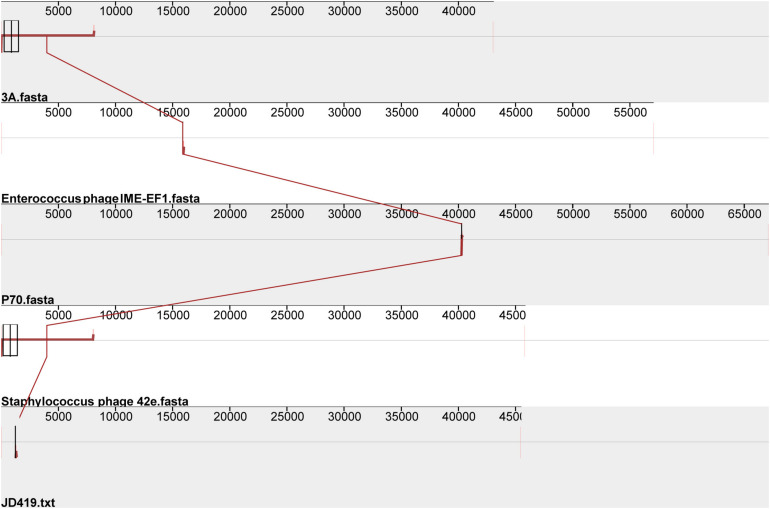
Comparative genomic analysis of *Staphylococcus* phage JD419 with other phages with similar morphology. *Staphylococcus* phage 3A, *Staphylococcus* phage 42e, *Enterococcus* phage IME-EF1, and *Listeria* phage P70.

**FIGURE 7:**
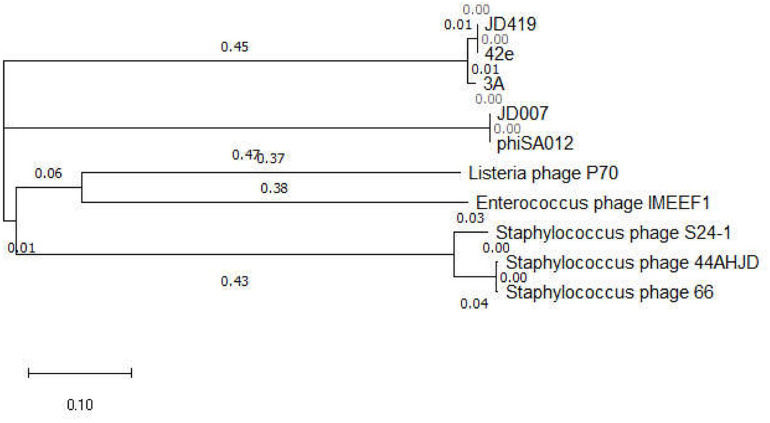
Phylogenetic tree of bacteriophages with similar morphology to JD419 and other phages infecting *S. aureus*, based on the major capsid protein sequences. Phage JD007 and phiSA012 belong to the family of *Myoviridae* infecting *S. aureus*. Phage S24-1, 44AHJD, and 66 belong to the family of *Podoviridae* infecting *S. aureus*. Phage JD419, 42e, and 3A belong to the family of *Siphoviridae* infecting *S. aureus.*

Interestingly, the three phages mentioned above with similar morphology infect Gram-positive bacteria and Gram-negative bacteria (Table 1). The phage IME-EF1 infects strains of *Enterococcus feacalis* (Zhang et al., 2013), phage P70 infects *Listeria monocytogenes* (Schmuki et al., 2012), phage 3A targets *S. aureus* (NC_007053.1), and phage 4CbK infects *Caulobacter crescentus* (Leonard et al., 1972). While it might be fair to assume that 3A is related to JD419, we find their identity to be at 95.98%, with a coverage of 82%; JD419 and 42e is 97.76%, however, with a coverage of 61%. When we compared the complete genome sequences with other morphologically similar phages, we did not find any close relatives among them (Figure 6). Interestingly, when analyzing distantly related phages of JD419, i.e., 42e, and 3A, the phages clustered in the same phylogenetic sub-branch when comparing their major capsid proteins only (Figure 7), which indicates that the phages are structurally related. It appears that the phages have acquired substantial differences in other genes required for infection and replication during evolution.

Phages pose the risk of contributing to the virulence of the target bacterial strains, introducing antibiotic resistance; the deployment of phages carrying these undesired genes in phage therapy should thus be avoided (Cui et al., 2017b). Therefore, it is essential to analyze phage genomes before using them in the clinic (Cui et al., 2017b). In the 45 kb genome of JD419, we predicted 65 ORFs, none of which encode any known virulence genes that confer antibiotic resistance. However, the results revealed putative genes encoding proteins like the CI repressor, an anti-repressor, and integrase. Such proteins are present in temperate phages. Using the phage genome analysis program PHACTS, JD419 was confidently classified as a temperate phage. Further indication that JD419 can undergo lysogeny is the observation that lysis is incomplete in some strains, as observed in the inhibition zones formed in some instances were turbid. In the case of other clinical isolates, the plaques appear transparent (identical to strictly lytic phages). When studying JD419 and the strain MR-84, we observe that the phage induces replication and lysis while it appears not to enter a dormant prophage state. Before using JD419 in therapy, all prophage-related genes should be removed by genetic engineering to avoid potential lysogeny and enhance bactericidal activity. Such approaches have been reported for *Mycobacterium* (Dedrick et al., 2019) as well as for *Salmonella* (Shin et al., 2014), while there are reports that showed the potential and possible use of temperate phages in therapy (Chung et al., 2012; Monteiro et al., 2019). Once the issue with lysogeny has been solved, the microbiological and biochemical characterization of JD419 indicates its potential to be used for phage therapy: JD419 has a broad host range tested on mostly multidrug-resistant clinical *S. aureus* strains.

Phage JD419 can infect approximately 44% of tested isolates that belong to various MLST types. However, bactericidal activity toward different strains varied significantly. As shown in Figures 3, 4, JD419 could perturb some strains’ growth, yet lysis was not fast and efficient. A high MOI was chosen to ensure that more than one phage infects each bacterial cell simultaneously in the study. Growth inhibition of the host strain at a MOI = 10 was observed. As shown in Figure 3A and Supplementary Figure 1A, it could more persistently inhibit N315 at MOI = 100 than at MOI = 10. While this effect might be absent in a genetically modified JD419 where prophage genes have been removed, the phage should still be tested *in vitro* for its bactericidal activity toward a target *S. aureus* strain before using it for therapy (Cui et al., 2019). Establishing a phage library containing different kinds of phages covering most strains would overcome the general disadvantage of phages being less practical than antibiotics (Ding et al., 2018). To avoid the emergence of phage-resistant bacteria, a highly efficient phage or a so-called phage cocktail of efficient phages could be used in phage therapy. In addition to their ability to infect and kill a target strain, phages should display stability to withstand the preparation and application, i.e., in clinical solutions or formulations for subsequent patient administration. Phage JD419 exhibits a fair degree of stability toward pH and temperature, indicating that the phage might be a suitable candidate as a therapeutic phage. The lability toward acidic pH values indicates that the phage might need to be encapsulated if it was to be delivered through the oral route (Loh et al., 2020b); however, *S. aureus* infections are usually observed in environments that display a pH close to neutral, easily tolerated by JD419, such as in the epidermis or the blood.

## Data Availability Statement

The datasets presented in this study can be found in online repositories. The names of the repository/repositories and accession number(s) can be found below: https://www.ncbi.nlm.nih.gov/genbank/, MT899504.

## Author Contributions

ZC and XG conceived and designed the experiments. ZC and TF performed the experiments. TF, BL, SL, ML, MS, and ZC analyzed the data. ZC, KD, BL, TF, YZ, and MS contributed reagents, materials, and analysis tools. TF, BL, SL, and ZC wrote the manuscript. All authors contributed to the article and approved the submitted version.

## Conflict of Interest

The authors declare that the research was conducted in the absence of any commercial or financial relationships that could be construed as a potential conflict of interest.

## Footnotes

1 http://ardb.cbcb.umd.edu/

2 http://mvirdb.llnl.gov/; http://www.mgc.ac.cn/VFs/main.htm
