# Supplementary material for: JD419, a Staphylococcus aureus Phage With a Unique Morphology and Broad Host Range
Source: Front Microbiol. 2021 Apr 22;12:602902. doi: 10.3389/fmicb.2021.602902 (PMC8100676; doi:10.3389/fmicb.2021.602902)
Supplement: Supplementary file 1 [file Image_1.pdf]

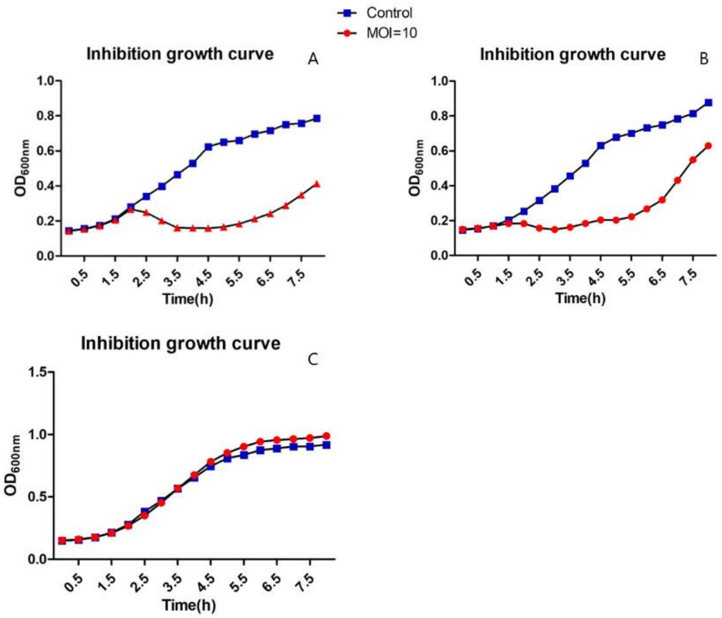

Fig.1suppl. **Inhibition assays of *Staphylococcus* phage JD419**. The x-axis represents the co-culture time of phage JD419 and different strains of *S.aureus*, and the y-axis represents the change in $_{OD600nm}$ in the mixture of *Staphylococcus* phage JD419 infecting different strains at the MOI=10. A represents strain N315; B represents strain MR-84; C represents strain MS-57.
