# Supplementary material for: JD419, a Staphylococcus aureus Phage With a Unique Morphology and Broad Host Range
Source: Front Microbiol. 2021 Apr 22;12:602902. doi: 10.3389/fmicb.2021.602902 (PMC8100676; doi:10.3389/fmicb.2021.602902)
Supplement: Supplementary file 2 [file Image_2.pdf]

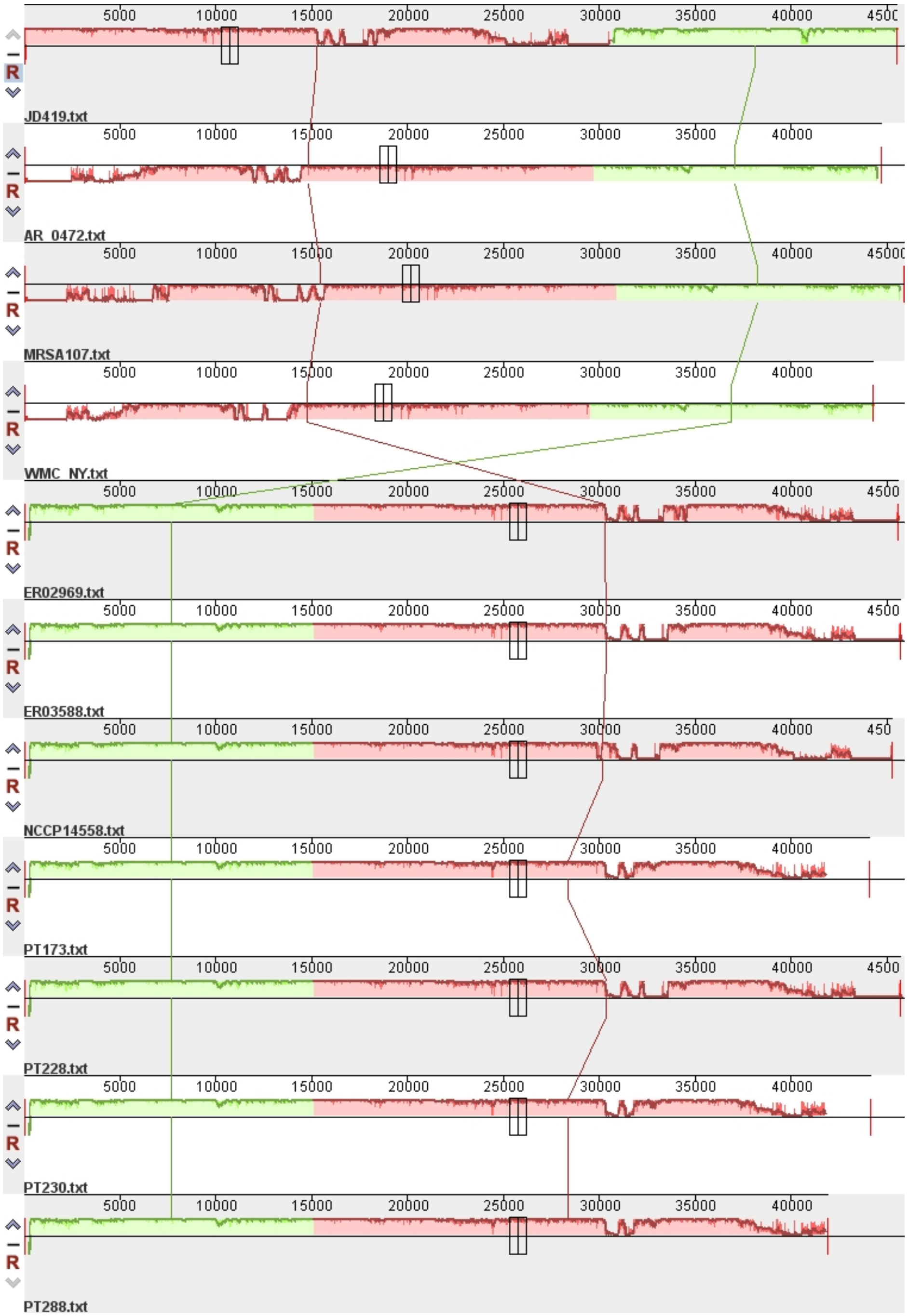

Suppl.Fig.2. Comparative genomic analysis of *Staphylococcus* phage JD419 with prophages predicted in the corresponding *S.aureus* strain. (Accession No. MRSA107: CP018629.1; PT288: CP049388.1; AR_0472: CP029649.1; PT228: CP049486.1; ER02969: CP030658.1; ER03588: CP030595.1; NCCP14558: CP013953.1; PT173: CP049580.1; PT230: CP049482.1; WMC_NY: CP063990.1).
